# *E*-[4-(β-d-Allopyranos­yloxy)phen­yl]-1-(4-chloro­phen­yl)prop-2-enone ethanol solvate

**DOI:** 10.1107/S1600536809006424

**Published:** 2009-02-28

**Authors:** Cong-ling Yang, Hua-ling Luo, Xiu-juan Yin, Ying Li, Shu-fan Yin

**Affiliations:** a College of Chemistry, Sichuan University, Chengdu 610064, People’s Republic of China

## Abstract

The title compound, C_21_H_21_ClO_7_·C_2_H_5_OH was synthesized by the condensation reaction between helicid [systematic name: 4-(β-d-allopyranos­yloxy)benzaldehyde] and 4-chloro­aceto­phen­one in ethanol. In the mol­ecular structure, the pyran­oside ring adopts a chair conformation. In the crystal structure, the molecules are linked by inter­molecular O—H⋯O hydrogen bonds involving the OH groups from the pyran­oside unit and from the ethanol solvent mol­ecule.

## Related literature

For helicid, see: Chen *et al.* (1981 ▶) and for its biological activity, see: Sha & Mao (1987 ▶). For the pharmacological activity of some helicid derivatives, see: Fan *et al.* (2007 ▶). 
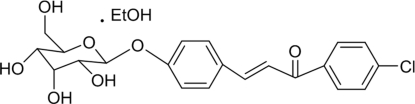


## Experimental

### 

#### Crystal data

C_21_H_21_ClO_7_·C_2_H_6_O
*M* *_r_* = 466.90
Monoclinic, 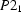

*a* = 11.000 (5) Å
*b* = 7.712 (3) Å
*c* = 13.213 (4) Å
β = 92.08 (2)°
*V* = 1120.2 (8) Å^3^
*Z* = 2
Mo *K*α radiation
μ = 0.22 mm^−1^
*T* = 292 K
0.48 × 0.44 × 0.36 mm

#### Data collection

Enraf–Nonius CAD-4 diffractometer
Absorption correction: for a sphere [*WinGX*; Farrugia, 1999 ▶)] *T*_min_ = 0.903, *T*_max_ = 0.926
2921 measured reflections
2815 independent reflections
2305 reflections with *I* > 2σ(*I*)
*R*_int_ = 0.008
3 standard reflections every 200 reflections intensity decay: 1.4%

#### Refinement

*R*[*F* ^2^ > 2σ(*F* ^2^)] = 0.048
*wR*(*F* ^2^) = 0.143
*S* = 1.10
2815 reflections
281 parameters
1 restraint
H-atom parameters constrained
Δρ_max_ = 0.43 e Å^−3^
Δρ_min_ = −0.30 e Å^−3^
Absolute structure: Flack (1983 ▶), 562 Friedel pairs
Flack parameter: 0.14 (12)

### 

Data collection: *DIFRAC* (Gabe & White, 1993 ▶); cell refinement: *DIFRAC*; data reduction: *NRCVAX* (Gabe *et al.*, 1989 ▶); program(s) used to solve structure: *SHELXS97* (Sheldrick, 2008 ▶); program(s) used to refine structure: *SHELXL97* (Sheldrick, 2008 ▶); molecular graphics: *ORTEP-3* (Farrugia, 1997 ▶); software used to prepare material for publication: *SHELXL97*.

## Supplementary Material

Crystal structure: contains datablocks global, I. DOI: 10.1107/S1600536809006424/bh2216sup1.cif

Structure factors: contains datablocks I. DOI: 10.1107/S1600536809006424/bh2216Isup2.hkl

Additional supplementary materials:  crystallographic information; 3D view; checkCIF report

## Figures and Tables

**Table 1:** Hydrogen-bond geometry (Å, °)

*D*—H⋯*A*	*D*—H	H⋯*A*	*D*⋯*A*	*D*—H⋯*A*
O2—H2*O*⋯O3^i^	0.82	2.02	2.809 (4)	160
O4—H4*O*⋯O2^ii^	0.82	1.88	2.694 (4)	171
O5—H5*O*⋯O8^ii^	0.82	1.97	2.696 (5)	148

Symmetry codes: (i) 
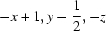
; (ii) 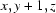.

**Fig. 1.**
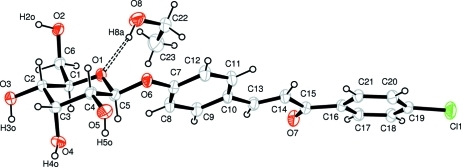
The molecular structure of (I), with displacement ellipsoids drawn at the 30% probability level.

## supplementary crystallographic information

### Comment

The natural compound helicid, 4-(β-D-allopyranosyloxy)benzaldehyde, which is extracted from the fruit of *Helicia nilagirica Beed* (Chen *et al.*, 1981) is a major active ingredient of Chinese herbal medicine. It has good biological effects on the central nervous system with a low toxicity (Sha & Mao, 1987). Some helicid derivatives have been reported with good pharmacological activities (Fan *et al.*, 2007). The title compound, a new helicid derivative, was synthesized *via* reaction of helicid and 4-chloroacetophenone, in good yield.

In the molecule of the title compound (Fig. 1), the average of C—C bond lengths in the six-membered pyranoside ring is 1.522 (5) Å. The average C(*sp*^3^)—O and C(*sp*^2^)—O bond lengths are 1.414 (5) and 1.392 (4) Å, respectively. The pyranoside ring adopts a chair conformation with hydroxyl group at C3 in axial position and the other substituents at C1, C2 and C4, in equatorial positions.

In the crystal packing, the molecules are connected by intermolecular O—H···.O hydrogen bonds (Table 1) involving O1 and O7 atoms and the hydroxyl groups in the main molecule and in the ethanol solvent molecule, forming a three-dimensional network.

### Experimental

To a solution of helicid (1.420 g, 5 mmol) in ethanol (20 ml), 10% aqueous solution of sodium hydroxide was added until helicid was dissolved completely. Then 4-chloroacetophenone (0.847 g, 5.5 mmol) was added dropwise, with the vessel placed in an ice bath. The reaction was monitored by TLC. After the reaction completed, the mixture was cooled to room temperature, and then neutralized with diluted hydrochloric acid. The solution was extracted three times with ethyl acetate, and the combined organic layers were dried with anhydrous Na_2_SO_4_, filtered, and evaporated *in vacuo* to get the crude product. The title compound was recrystallized twice from ethanol, and colourless single crystals were finally obtained by slow evaporation of an ethanol solution, at room temperature.

### Refinement

H atoms were positioned geometrically (C—H = 0.93–0.98 Å, O—H = 0.82 Å) and refined using a riding model, with *U*_iso_(H) = 1.2*U*_eq_ (methylene C, aromatic C) or *U*_iso_(H) = 1.5*U*_eq_ (O). The absolute configuration was determined by refinement of a Flack parameter, based on 562 measured Friedel pairs (Flack, 1983), and is in agreement with the expected configuration from the synthetic route.

### Crystal data

**Table:**

C_21_H_21_ClO_7_·C_2_H_6_O	*F*(000) = 492
*M**_r_* = 466.90	*D*_x_ = 1.384 Mg m^−^^3^
Monoclinic, *P*2_1_	Mo *K*α radiation, λ = 0.71073 Å
Hall symbol: P 2yb	Cell parameters from 19 reflections
*a* = 11.000 (5) Å	θ = 4.5–7.4°
*b* = 7.712 (3) Å	µ = 0.22 mm^−^^1^
*c* = 13.213 (4) Å	*T* = 292 K
β = 92.08 (2)°	Block, colourless
*V* = 1120.2 (8) Å^3^	0.48 × 0.44 × 0.36 mm
*Z* = 2	

### Data collection

**Table:**

Enraf–Nonius CAD-4 diffractometer	2305 reflections with *I* > 2σ(*I*)
Radiation source: fine-focus sealed tube	*R*_int_ = 0.008
graphite	θ_max_ = 25.5°, θ_min_ = 1.5°
ω/–2θ scans	*h* = −13→13
Absorption correction: for a sphere [*WinGX*; Farrugia, 1999)]	*k* = −9→9
*T*_min_ = 0.903, *T*_max_ = 0.926	*l* = −15→15
2921 measured reflections	3 standard reflections every 200 reflections
2815 independent reflections	intensity decay: 1.4%

### Refinement

**Table:**

Refinement on *F*^2^	Hydrogen site location: inferred from neighbouring sites
Least-squares matrix: full	H-atom parameters constrained
*R*[*F*^2^ > 2σ(*F*^2^)] = 0.048	*w* = 1/[σ^2^(*F*_o_^2^) + (0.099*P*)^2^ + 0.0279*P*] where *P* = (*F*_o_^2^ + 2*F*_c_^2^)/3
*wR*(*F*^2^) = 0.143	(Δ/σ)_max_ = 0.001
*S* = 1.10	Δρ_max_ = 0.43 e Å^−^^3^
2815 reflections	Δρ_min_ = −0.30 e Å^−^^3^
281 parameters	Extinction correction: *SHELXL97* (Sheldrick, 2008), Fc^*^=kFc[1+0.001xFc^2^λ^3^/sin(2θ)]^-1/4^
1 restraint	Extinction coefficient: 0.056 (7)
Primary atom site location: structure-invariant direct methods	Absolute structure: Flack (1983), 562 Friedel pairs
Secondary atom site location: difference Fourier map	Flack parameter: 0.14 (12)

### Fractional atomic coordinates and isotropic or equivalent isotropic displacement parameters (Å^2^)

**Table:**

	*x*	*y*	*z*	*U*_iso_*/*U*_eq_	
Cl1	1.19712 (12)	0.6652 (2)	1.29357 (8)	0.0730 (4)	
O1	0.6580 (2)	0.7216 (3)	0.27293 (17)	0.0385 (6)	
O2	0.5789 (3)	0.4472 (3)	0.1477 (2)	0.0478 (7)	
H2O	0.5624	0.4470	0.0867	0.072*	
O3	0.4614 (2)	0.8598 (4)	0.05711 (19)	0.0469 (7)	
H3O	0.4201	0.9331	0.0848	0.070*	
O4	0.5518 (3)	1.1087 (3)	0.1920 (2)	0.0452 (7)	
H4O	0.5665	1.2117	0.1830	0.068*	
O5	0.8179 (2)	1.1280 (4)	0.2200 (2)	0.0481 (7)	
H5O	0.7732	1.2009	0.2443	0.072*	
O6	0.8220 (2)	0.8419 (4)	0.35453 (16)	0.0423 (6)	
O7	0.7388 (3)	0.5708 (5)	0.9598 (2)	0.0564 (8)	
C1	0.5463 (3)	0.7382 (5)	0.2130 (2)	0.0353 (8)	
H1	0.4880	0.8055	0.2513	0.042*	
C2	0.5718 (4)	0.8348 (5)	0.1143 (3)	0.0376 (8)	
H2	0.6262	0.7634	0.0745	0.045*	
C3	0.6344 (3)	1.0062 (5)	0.1375 (3)	0.0386 (8)	
H3	0.6536	1.0646	0.0742	0.046*	
C4	0.7517 (3)	0.9739 (5)	0.2010 (3)	0.0373 (8)	
H4	0.8030	0.8947	0.1633	0.045*	
C5	0.7157 (3)	0.8832 (5)	0.2974 (3)	0.0379 (8)	
H5	0.6621	0.9570	0.3365	0.046*	
C6	0.4973 (4)	0.5577 (5)	0.1971 (3)	0.0431 (9)	
H6A	0.4793	0.5079	0.2623	0.052*	
H6B	0.4217	0.5640	0.1571	0.052*	
C7	0.8102 (3)	0.7956 (5)	0.4555 (3)	0.0401 (9)	
C8	0.6991 (3)	0.7808 (6)	0.4999 (3)	0.0468 (10)	
H8	0.6269	0.8012	0.4631	0.056*	
C9	0.6987 (3)	0.7340 (6)	0.6024 (3)	0.0475 (9)	
H9	0.6247	0.7247	0.6338	0.057*	
C10	0.8041 (3)	0.7015 (5)	0.6579 (3)	0.0423 (9)	
C11	0.9136 (4)	0.7145 (6)	0.6094 (3)	0.0497 (10)	
H11	0.9861	0.6905	0.6450	0.060*	
C12	0.9159 (4)	0.7624 (6)	0.5098 (3)	0.0490 (10)	
H12	0.9901	0.7725	0.4787	0.059*	
C13	0.7923 (3)	0.6580 (6)	0.7646 (3)	0.0469 (9)	
H13	0.7131	0.6442	0.7855	0.056*	
C14	0.8794 (3)	0.6351 (6)	0.8365 (3)	0.0454 (9)	
H14	0.9610	0.6407	0.8206	0.054*	
C15	0.8446 (3)	0.6010 (5)	0.9412 (3)	0.0409 (9)	
C16	0.9379 (3)	0.6058 (5)	1.0255 (3)	0.0379 (8)	
C17	0.9023 (4)	0.5637 (5)	1.1225 (3)	0.0444 (9)	
H17	0.8234	0.5253	1.1321	0.053*	
C18	0.9827 (4)	0.5781 (6)	1.2042 (3)	0.0480 (10)	
H18	0.9586	0.5490	1.2688	0.058*	
C19	1.0986 (4)	0.6360 (5)	1.1897 (3)	0.0453 (9)	
C20	1.1381 (4)	0.6725 (6)	1.0948 (3)	0.0489 (10)	
H20	1.2180	0.7066	1.0859	0.059*	
C21	1.0571 (3)	0.6578 (5)	1.0122 (3)	0.0420 (9)	
H21	1.0829	0.6830	0.9475	0.050*	
O8	0.7509 (3)	0.3817 (5)	0.3469 (3)	0.0700 (10)*	
H8A	0.6815	0.3984	0.3242	0.105*	
C22	0.7467 (4)	0.3290 (7)	0.4506 (3)	0.0590 (11)*	
H22A	0.7872	0.4154	0.4931	0.071*	
H22B	0.7903	0.2204	0.4596	0.071*	
C23	0.6220 (6)	0.3068 (9)	0.4826 (5)	0.0845 (16)*	
H23A	0.5798	0.2276	0.4377	0.127*	
H23B	0.5812	0.4168	0.4810	0.127*	
H23C	0.6231	0.2614	0.5503	0.127*	

### Atomic displacement parameters (Å^2^)

**Table:**

	*U*^11^	*U*^22^	*U*^33^	*U*^12^	*U*^13^	*U*^23^
Cl1	0.0730 (8)	0.1004 (10)	0.0442 (6)	0.0021 (8)	−0.0177 (5)	−0.0029 (6)
O1	0.0469 (14)	0.0345 (11)	0.0336 (12)	0.0010 (12)	−0.0078 (11)	0.0031 (11)
O2	0.0706 (19)	0.0373 (14)	0.0350 (14)	0.0064 (14)	−0.0040 (14)	−0.0004 (11)
O3	0.0485 (16)	0.0526 (16)	0.0388 (14)	0.0065 (14)	−0.0115 (12)	0.0005 (13)
O4	0.0499 (16)	0.0336 (13)	0.0525 (16)	0.0062 (12)	0.0054 (12)	0.0011 (12)
O5	0.0461 (15)	0.0473 (15)	0.0513 (16)	−0.0075 (13)	0.0087 (12)	−0.0051 (13)
O6	0.0363 (13)	0.0620 (16)	0.0284 (12)	0.0025 (13)	−0.0022 (10)	0.0014 (12)
O7	0.0444 (16)	0.084 (2)	0.0410 (14)	−0.0089 (16)	0.0028 (12)	0.0011 (15)
C1	0.0376 (19)	0.0380 (17)	0.0299 (17)	0.0017 (15)	−0.0021 (15)	0.0000 (15)
C2	0.045 (2)	0.0357 (17)	0.0313 (17)	0.0040 (17)	−0.0020 (16)	0.0036 (16)
C3	0.048 (2)	0.0377 (18)	0.0303 (17)	0.0028 (17)	0.0023 (15)	0.0054 (15)
C4	0.042 (2)	0.0375 (18)	0.0325 (18)	0.0025 (16)	0.0023 (15)	−0.0031 (15)
C5	0.038 (2)	0.0394 (19)	0.0358 (19)	−0.0007 (16)	−0.0045 (16)	−0.0031 (15)
C6	0.047 (2)	0.0405 (19)	0.042 (2)	−0.0029 (18)	−0.0032 (17)	0.0031 (16)
C7	0.0409 (19)	0.047 (2)	0.0323 (18)	0.0004 (18)	−0.0026 (15)	−0.0035 (15)
C8	0.0351 (19)	0.070 (3)	0.0353 (18)	0.005 (2)	−0.0038 (15)	0.0006 (18)
C9	0.0367 (19)	0.070 (3)	0.0360 (19)	0.0027 (19)	0.0038 (16)	0.001 (2)
C10	0.042 (2)	0.054 (2)	0.0309 (17)	0.0017 (19)	−0.0012 (15)	0.0003 (17)
C11	0.040 (2)	0.072 (3)	0.0367 (19)	0.003 (2)	−0.0089 (16)	0.004 (2)
C12	0.040 (2)	0.070 (3)	0.0373 (19)	−0.001 (2)	0.0023 (16)	−0.0004 (19)
C13	0.045 (2)	0.061 (2)	0.0337 (18)	0.001 (2)	−0.0011 (16)	0.0002 (19)
C14	0.042 (2)	0.061 (2)	0.0332 (18)	−0.004 (2)	0.0007 (16)	0.0007 (18)
C15	0.042 (2)	0.046 (2)	0.0349 (18)	−0.0024 (17)	0.0034 (16)	−0.0016 (16)
C16	0.046 (2)	0.0378 (18)	0.0298 (17)	−0.0009 (17)	0.0019 (15)	0.0024 (15)
C17	0.043 (2)	0.051 (2)	0.040 (2)	0.0053 (18)	0.0057 (16)	0.0038 (17)
C18	0.058 (2)	0.056 (2)	0.0296 (18)	0.009 (2)	0.0033 (17)	0.0043 (17)
C19	0.055 (2)	0.047 (2)	0.0336 (18)	0.003 (2)	−0.0049 (16)	0.0001 (17)
C20	0.045 (2)	0.056 (3)	0.045 (2)	−0.006 (2)	0.0004 (17)	0.003 (2)
C21	0.046 (2)	0.048 (2)	0.0321 (17)	−0.0023 (19)	0.0013 (15)	0.0031 (17)

### Geometric parameters (Å, °)

**Table:**

Cl1—C19	1.732 (4)	C9—C10	1.372 (5)
O1—C5	1.431 (4)	C9—H9	0.9300
O1—C1	1.443 (4)	C10—C11	1.389 (5)
O2—C6	1.414 (5)	C10—C13	1.460 (5)
O2—H2O	0.8200	C11—C12	1.368 (6)
O3—C2	1.420 (4)	C11—H11	0.9300
O3—H3O	0.8200	C12—H12	0.9300
O4—C3	1.420 (5)	C13—C14	1.336 (5)
O4—H4O	0.8200	C13—H13	0.9300
O5—C4	1.412 (5)	C14—C15	1.473 (5)
O5—H5O	0.8200	C14—H14	0.9300
O6—C7	1.392 (4)	C15—C16	1.488 (5)
O6—C5	1.404 (4)	C16—C21	1.389 (5)
O7—C15	1.221 (5)	C16—C17	1.392 (5)
C1—C6	1.505 (5)	C17—C18	1.376 (5)
C1—C2	1.537 (5)	C17—H17	0.9300
C1—H1	0.9800	C18—C19	1.371 (6)
C2—C3	1.516 (5)	C18—H18	0.9300
C2—H2	0.9800	C19—C20	1.371 (6)
C3—C4	1.533 (5)	C20—C21	1.388 (5)
C3—H3	0.9800	C20—H20	0.9300
C4—C5	1.519 (5)	C21—H21	0.9300
C4—H4	0.9800	O8—C22	1.431 (5)
C5—H5	0.9800	O8—H8A	0.8200
C6—H6A	0.9700	C22—C23	1.460 (7)
C6—H6B	0.9700	C22—H22A	0.9700
C7—C12	1.368 (5)	C22—H22B	0.9700
C7—C8	1.379 (5)	C23—H23A	0.9600
C8—C9	1.402 (5)	C23—H23B	0.9600
C8—H8	0.9300	C23—H23C	0.9600
			
C5—O1—C1	114.1 (3)	C8—C9—H9	119.0
C6—O2—H2O	109.5	C9—C10—C11	118.1 (3)
C2—O3—H3O	109.5	C9—C10—C13	117.1 (3)
C3—O4—H4O	109.5	C11—C10—C13	124.8 (3)
C4—O5—H5O	109.5	C12—C11—C10	120.7 (3)
C7—O6—C5	117.9 (3)	C12—C11—H11	119.7
O1—C1—C6	106.7 (3)	C10—C11—H11	119.7
O1—C1—C2	109.3 (3)	C11—C12—C7	120.7 (4)
C6—C1—C2	113.9 (3)	C11—C12—H12	119.7
O1—C1—H1	108.9	C7—C12—H12	119.7
C6—C1—H1	108.9	C14—C13—C10	129.1 (4)
C2—C1—H1	108.9	C14—C13—H13	115.5
O3—C2—C3	111.2 (3)	C10—C13—H13	115.5
O3—C2—C1	109.8 (3)	C13—C14—C15	119.1 (3)
C3—C2—C1	110.3 (3)	C13—C14—H14	120.4
O3—C2—H2	108.5	C15—C14—H14	120.4
C3—C2—H2	108.5	O7—C15—C14	120.4 (3)
C1—C2—H2	108.5	O7—C15—C16	119.4 (3)
O4—C3—C2	107.1 (3)	C14—C15—C16	120.2 (3)
O4—C3—C4	110.7 (3)	C21—C16—C17	118.9 (3)
C2—C3—C4	109.6 (3)	C21—C16—C15	122.8 (3)
O4—C3—H3	109.8	C17—C16—C15	118.3 (3)
C2—C3—H3	109.8	C18—C17—C16	120.7 (4)
C4—C3—H3	109.8	C18—C17—H17	119.7
O5—C4—C5	112.7 (3)	C16—C17—H17	119.7
O5—C4—C3	112.3 (3)	C19—C18—C17	119.4 (4)
C5—C4—C3	107.1 (3)	C19—C18—H18	120.3
O5—C4—H4	108.2	C17—C18—H18	120.3
C5—C4—H4	108.2	C18—C19—C20	121.5 (4)
C3—C4—H4	108.2	C18—C19—Cl1	119.3 (3)
O6—C5—O1	106.1 (3)	C20—C19—Cl1	119.2 (3)
O6—C5—C4	108.6 (3)	C19—C20—C21	119.2 (4)
O1—C5—C4	109.8 (3)	C19—C20—H20	120.4
O6—C5—H5	110.8	C21—C20—H20	120.4
O1—C5—H5	110.8	C20—C21—C16	120.3 (4)
C4—C5—H5	110.8	C20—C21—H21	119.8
O2—C6—C1	113.1 (3)	C16—C21—H21	119.8
O2—C6—H6A	109.0	C22—O8—H8A	109.5
C1—C6—H6A	109.0	O8—C22—C23	112.0 (4)
O2—C6—H6B	109.0	O8—C22—H22A	109.2
C1—C6—H6B	109.0	C23—C22—H22A	109.2
H6A—C6—H6B	107.8	O8—C22—H22B	109.2
C12—C7—C8	120.7 (3)	C23—C22—H22B	109.2
C12—C7—O6	116.4 (3)	H22A—C22—H22B	107.9
C8—C7—O6	122.9 (3)	C22—C23—H23A	109.5
C7—C8—C9	117.8 (3)	C22—C23—H23B	109.5
C7—C8—H8	121.1	H23A—C23—H23B	109.5
C9—C8—H8	121.1	C22—C23—H23C	109.5
C10—C9—C8	122.0 (4)	H23A—C23—H23C	109.5
C10—C9—H9	119.0	H23B—C23—H23C	109.5
			
C5—O1—C1—C6	−178.6 (3)	C7—C8—C9—C10	−0.7 (7)
C5—O1—C1—C2	57.8 (4)	C8—C9—C10—C11	−0.6 (7)
O1—C1—C2—O3	−177.0 (3)	C8—C9—C10—C13	178.6 (4)
C6—C1—C2—O3	63.7 (4)	C9—C10—C11—C12	1.5 (7)
O1—C1—C2—C3	−54.1 (4)	C13—C10—C11—C12	−177.6 (4)
C6—C1—C2—C3	−173.4 (3)	C10—C11—C12—C7	−1.1 (7)
O3—C2—C3—O4	58.7 (4)	C8—C7—C12—C11	−0.2 (7)
C1—C2—C3—O4	−63.3 (4)	O6—C7—C12—C11	−179.5 (4)
O3—C2—C3—C4	178.9 (3)	C9—C10—C13—C14	−173.8 (4)
C1—C2—C3—C4	56.8 (4)	C11—C10—C13—C14	5.2 (7)
O4—C3—C4—O5	−65.5 (4)	C10—C13—C14—C15	176.6 (4)
C2—C3—C4—O5	176.5 (3)	C13—C14—C15—O7	9.8 (6)
O4—C3—C4—C5	58.7 (4)	C13—C14—C15—C16	−169.3 (4)
C2—C3—C4—C5	−59.2 (4)	O7—C15—C16—C21	−172.7 (4)
C7—O6—C5—O1	−76.9 (4)	C14—C15—C16—C21	6.4 (6)
C7—O6—C5—C4	165.2 (3)	O7—C15—C16—C17	4.3 (5)
C1—O1—C5—O6	−179.6 (3)	C14—C15—C16—C17	−176.6 (4)
C1—O1—C5—C4	−62.5 (4)	C21—C16—C17—C18	1.9 (6)
O5—C4—C5—O6	−59.6 (4)	C15—C16—C17—C18	−175.2 (3)
C3—C4—C5—O6	176.3 (3)	C16—C17—C18—C19	0.5 (6)
O5—C4—C5—O1	−175.2 (3)	C17—C18—C19—C20	−2.9 (6)
C3—C4—C5—O1	60.8 (3)	C17—C18—C19—Cl1	176.6 (3)
O1—C1—C6—O2	−58.8 (4)	C18—C19—C20—C21	2.9 (6)
C2—C1—C6—O2	61.8 (4)	Cl1—C19—C20—C21	−176.6 (3)
C5—O6—C7—C12	−177.7 (3)	C19—C20—C21—C16	−0.4 (6)
C5—O6—C7—C8	3.1 (6)	C17—C16—C21—C20	−2.0 (6)
C12—C7—C8—C9	1.1 (6)	C15—C16—C21—C20	175.1 (4)
O6—C7—C8—C9	−179.7 (4)		

### Hydrogen-bond geometry (Å, °)

**Table:**

*D*—H···*A*	*D*—H	H···*A*	*D*···*A*	*D*—H···*A*
O2—H2O···O3^i^	0.82	2.02	2.809 (4)	160
O4—H4O···O2^ii^	0.82	1.88	2.694 (4)	171
O5—H5O···O8^ii^	0.82	1.97	2.696 (5)	148
O3—H3O···O7^iii^	0.82	2.11	2.741 (4)	134
O3—H3O···O4	0.82	2.41	2.779 (4)	109
O8—H8A···O1	0.82	2.59	2.966 (5)	109

Symmetry codes: (i) −*x*+1, *y*−1/2, −*z*; (ii) *x*, *y*+1, *z*; (iii) −*x*+1, *y*+1/2, −*z*+1.
